# Supplementary material for: Association between oral microbiome and seven types of cancers in East Asian population: a two-sample Mendelian randomization analysis
Source: Front Mol Biosci. 2023 Nov 21;10:1327893. doi: 10.3389/fmolb.2023.1327893 (PMC10702768; doi:10.3389/fmolb.2023.1327893)
Supplement: Supplementary file 1 [file DataSheet1.ZIP › Supplementary Materials/MR plots of saliva/saliva═╝/Lung Cancer/pheno.2528_to_lung cancer_leave_one_out.pdf]

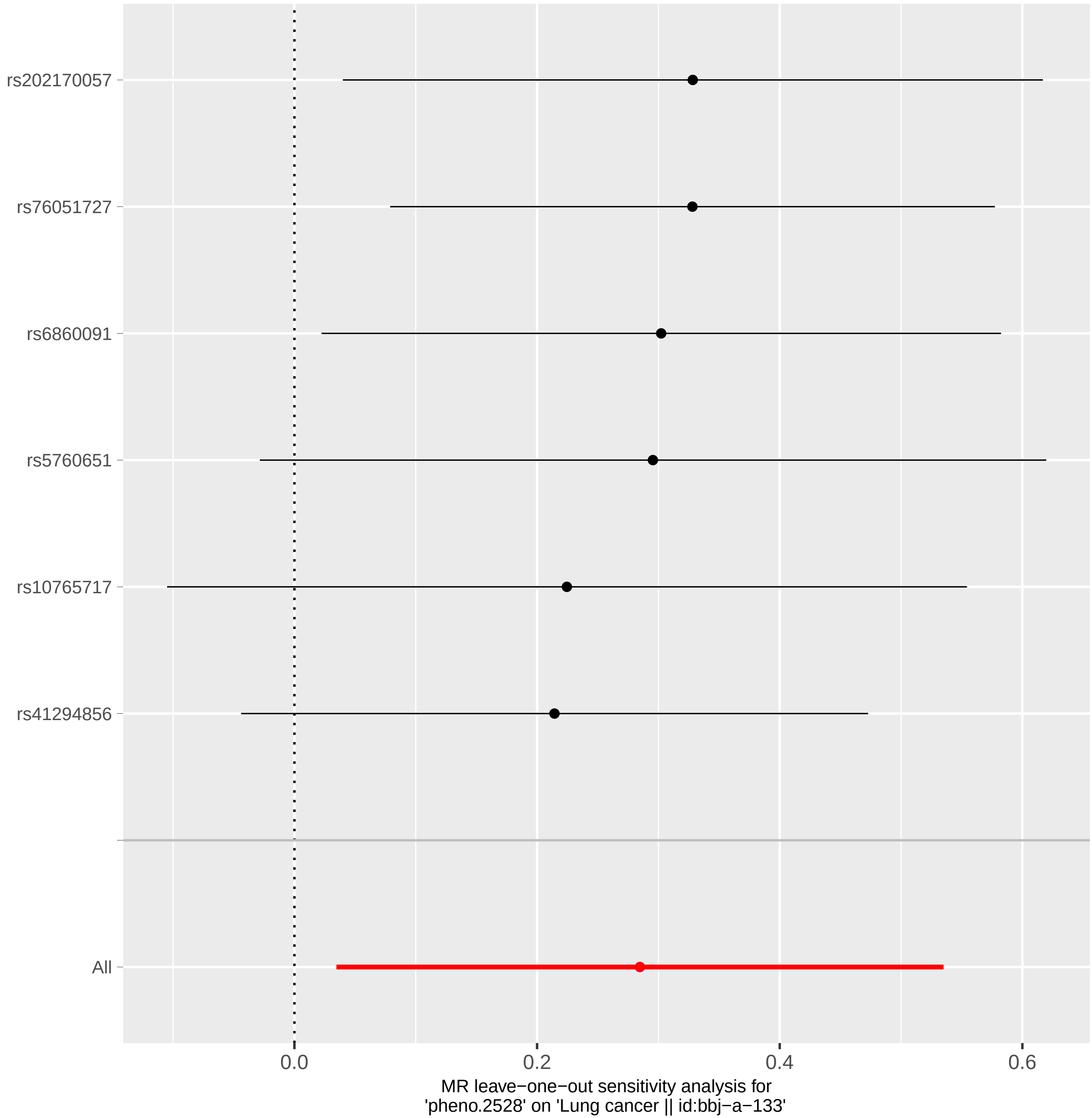
rs202170057
rs76051727
rs6860091
rs5760651
rs10765717
rs41294856
All
0.0
0.2
0.4
0.6
MR leave−one−out sensitivity analysis for
'pheno.2528' on 'Lung cancer || id:bbj−a−133'
